# 1-(2′,4′-Difluoro­biphenyl-4-yl)ethanone

**DOI:** 10.1107/S1600536808028596

**Published:** 2008-09-13

**Authors:** Meng-Ping Guo, Ji-Hua Deng, Qiao-Chu Zhang, Hui-Rui Guo, Lin Yuan

**Affiliations:** a Institute of Coordination Catalysis, Yichun University, Yichun, Jiangxi, 336000, People’s Republic of China; b College of Chemistry and Bio-engineering, Yichun University, Yichun, Jiangxi, 336000, People’s Republic of China

## Abstract

In the crystal structure of the title compound, C_14_H_10_F_2_O, the dihedral angles between the benzene rings in the two crystallographically independent mol­ecules are 46.9 (2) and 47.6 (2)°. The mol­ecules are linked into dimers by C—H⋯F inter­actions and these dimers are further stacked into columns along the *b* axis by π–π inter­actions between the benzene rings [centroid–centroid distance = 3.8221 Å; the dihedral angle between the planes of these rings is 4.87 (2)°]. In addition, C—F⋯π interactions also contribute to the crystal packing (C⋯centroid distance = 3.5919 Å).

## Related literature

For general background, see: William & Ruyle (1973 ▶). For related structures, see:Kuchar *et al.* (1997 ▶); Jegorov *et al.* (1995 ▶, 1997 ▶).
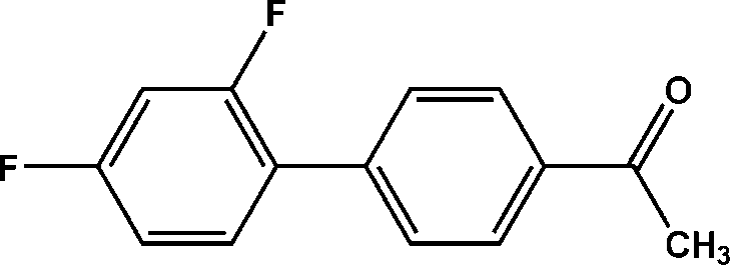


## Experimental

### 

#### Crystal data

C_14_H_10_F_2_O
*M* *_r_* = 232.22
Orthorhombic, 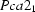

*a* = 13.092 (7) Å
*b* = 6.102 (3) Å
*c* = 27.719 (15) Å
*V* = 2214 (2) Å^3^
*Z* = 8
Mo *K*α radiation
μ = 0.11 mm^−1^
*T* = 293 (2) K
0.46 × 0.32 × 0.25 mm

#### Data collection

Bruker P4 diffractometer
Absorption correction: none
15497 measured reflections
2576 independent reflections
2219 reflections with *I* > 2σ(*I*)
*R*_int_ = 0.033

#### Refinement

*R*[*F* ^2^ > 2σ(*F* ^2^)] = 0.066
*wR*(*F* ^2^) = 0.207
*S* = 1.13
2576 reflections
309 parameters
1 restraint
H-atom parameters constrained
Δρ_max_ = 0.80 e Å^−3^
Δρ_min_ = −0.33 e Å^−3^

### 

Data collection: *SMART* (Bruker, 2004 ▶); cell refinement: *SAINT* (Bruker, 2004 ▶); data reduction: *SAINT*; program(s) used to solve structure: *SHELXS97* (Sheldrick, 2008 ▶); program(s) used to refine structure: *SHELXL97* (Sheldrick, 2008 ▶); molecular graphics: *SHELXTL* (Sheldrick, 2008 ▶); software used to prepare material for publication: *SHELXTL*.

## Supplementary Material

Crystal structure: contains datablocks gmp, I. DOI: 10.1107/S1600536808028596/nc2113sup1.cif

Structure factors: contains datablocks I. DOI: 10.1107/S1600536808028596/nc2113Isup2.hkl

Additional supplementary materials:  crystallographic information; 3D view; checkCIF report

## Figures and Tables

**Table 1:** Hydrogen-bond geometry (Å, °)

*D*—H⋯*A*	*D*—H	H⋯*A*	*D*⋯*A*	*D*—H⋯*A*
C12—H12*A*⋯F2^i^	0.93	2.46	3.369 (6)	167
C22—H22⋯F4^ii^	0.93	2.40	3.318 (6)	167

Symmetry codes: (i) 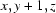; (ii) 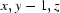.

**Table 2:** C—F⋯π interactions (Å, °)

C—F⋯*Cg*^*a*^	F⋯*Cg*	C⋯*Cg*	γ^*b*^	C—F⋯*Cg*
C1—F1⋯*Cg*(C7→C12)^iii^	3.8358	3.5919	27.36	69
C15—F3⋯*Cg*(C21→C26)^iv^	3.8814	3.5966	27.14	68

Notes: *Cg* ^a^ = centre of gravity of the six-membered ring. γ^b^ = angle defined by a line connecting the centre of gravity of the six-membered ring with H atom and the normal to the six-membered ring. Symmetry codes: (iii) 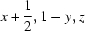; (IV) 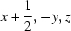.

**Table 3:** π⋯π interactions (Å, °)

π⋯π contacts	*Cg*⋯*Cg*(Å)	α^*a*^(°)	β^*b*^(°)	*Cg*⋯Plane(Å)
*Cg*(C1→C6)⋯*Cg*(C7→C12)^iii^	3.8221	4.87	27.32	3.616
*Cg*(C15→C20)⋯*Cg*(C21→C26)^iv^	3.8284	5.68	23.57	3.642

Notes: α^a^ = angle between planes of two aromatic rings. β^b^ = angle between *Cg*⋯*Cg* line and normal to the plane of the first aromatic ring. Symmetry codes: (iii) 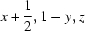; (IV) 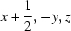.

**Fig. 1.**
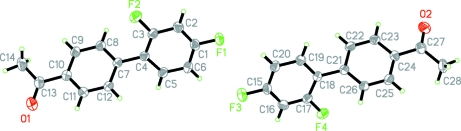
Crystal structure of the title compound showing 30% probability displacement ellipsoids and the atom-labeling scheme.

## supplementary crystallographic information

### Comment

The crystal structure of the title compound was determined as a part of a project on the synthesis of 1-(2',4'-difluorobiphenyl-4-yl)ethanone, which have excellent activity against various bacteria, antifebrile and abirritation (William *et al.*, 1973; Kuchar *et al.*, 1997; Jegorov, Husak *et al.*, 1997; Jegorov, Sedmera *et al.*, 1995). The title compound is an intermediate in this synthesis.

The asymmetric unit of (I) contains two crystallographically indepedent molecules of the same stereochemical configuration (Fig. 1). The dihedral angle between aromatic ring C1-C6 and C7-C12 amount to 46.8 (2) ° and between ring C15-C20 and C21-C26 it is 47.6 (2) ° (Fig. 1). The molecules are connected into dimers via C—H···F interactions (Table 1). There also exist π—π stacking interactions between the benzene rings of adjacent molecules. The distances of the centroids of the rings C1-C6 and C7-C12 as well as rings C15-C20 and C21-C26) amount to 3.8821 and 3.8284 /%A, respectively. In the direction of the b-axis, the molecules shows a herringbone like arrangement.

### Experimental

0.1892 g (1 mmol) 1-(4-bromophenyl)ethanone, 0.2385 g (1.5 mmol) 2,4-difluorophenylboronic acid, 0.2123 g (2 mmol) Na_2_CO_3_ and 0.2255 g (1 mmol) Pd(OAc)_2_ were dissolved in a water-acetone mixture (1:1 *v*/*v*; 50 ml). After stirring for 20 min at 308 K, the mixture were extracted using diethylether for four times. Then the resultant diethylether solution were dried over magnesium sulfate and concentrated *in vacuo*. Well shaped light yellow crystals suitable for X-ray structure analysis were obtained by recrystalling the crude product from ethanol.

### Refinement

Hydrogen atoms attached to carbon atoms were positioned with idealized geometry and were refined isotropic with *U*_iso_(H) = 1.2*U*_eq_(C) using a riding model with C—H = 0.93 Å . The absolute structure cannot be determined because no strong anomalous scattering atoms are present. Therefore, the Friedel opposites have been merged in the refinement.

### Crystal data

**Table:**

C_14_H_10_F_2_O	*F*(000) = 960
*M**_r_* = 232.22	*D*_x_ = 1.393 Mg m^−^^3^
Orthorhombic, *P**c**a*2_1_	Mo *K*α radiation, λ = 0.71073 Å
Hall symbol: P 2c -2ac	Cell parameters from 2398 reflections
*a* = 13.092 (7) Å	θ = 2.9–27.5°
*b* = 6.102 (3) Å	µ = 0.11 mm^−^^1^
*c* = 27.719 (15) Å	*T* = 293 K
*V* = 2214 (2) Å^3^	Block, colorless
*Z* = 8	0.46 × 0.32 × 0.25 mm

### Data collection

**Table:**

Bruker P4 diffractometer	2219 reflections with *I* > 2σ(*I*)
Radiation source: fine-focus sealed tube	*R*_int_ = 0.033
graphite	θ_max_ = 27.5°, θ_min_ = 2.9°
ω scans	*h* = −16→16
15497 measured reflections	*k* = −7→7
2576 independent reflections	*l* = −35→26

### Refinement

**Table:**

Refinement on *F*^2^	Primary atom site location: structure-invariant direct methods
Least-squares matrix: full	Secondary atom site location: difference Fourier map
*R*[*F*^2^ > 2σ(*F*^2^)] = 0.066	Hydrogen site location: inferred from neighbouring sites
*wR*(*F*^2^) = 0.207	H-atom parameters constrained
*S* = 1.13	*w* = 1/[σ^2^(*F*_o_^2^) + (0.135*P*)^2^ + 0.237*P*] where *P* = (*F*_o_^2^ + 2*F*_c_^2^)/3
2576 reflections	(Δ/σ)_max_ = 0.045
309 parameters	Δρ_max_ = 0.80 e Å^−^^3^
1 restraint	Δρ_min_ = −0.33 e Å^−^^3^

### Special details

**Table:**

Geometry. All e.s.d.'s (except the e.s.d. in the dihedral angle between two l.s. planes) are estimated using the full covariance matrix. The cell e.s.d.'s are taken into account individually in the estimation of e.s.d.'s in distances, angles and torsion angles; correlations between e.s.d.'s in cell parameters are only used when they are defined by crystal symmetry. An approximate (isotropic) treatment of cell e.s.d.'s is used for estimating e.s.d.'s involving l.s. planes.
Refinement. Refinement of *F*^2^ against ALL reflections. The weighted *R*-factor *wR* and goodness of fit *S* are based on *F*^2^, conventional *R*-factors *R* are based on *F*, with *F* set to zero for negative *F*^2^. The threshold expression of *F*^2^ > σ(*F*^2^) is used only for calculating *R*-factors(gt) *etc*. and is not relevant to the choice of reflections for refinement. *R*-factors based on *F*^2^ are statistically about twice as large as those based on *F*, and *R*- factors based on ALL data will be even larger.

### Fractional atomic coordinates and isotropic or equivalent isotropic displacement parameters (Å^2^)

**Table:**

	*x*	*y*	*z*	*U*_iso_*/*U*_eq_	
F1	0.3975 (2)	0.4954 (5)	1.02287 (16)	0.0734 (10)	
F2	0.7101 (3)	0.2074 (5)	0.97293 (14)	0.0819 (10)	
O1	1.0793 (3)	0.8318 (7)	0.83509 (19)	0.0858 (13)	
C1	0.4849 (4)	0.5145 (8)	0.9977 (2)	0.0567 (13)	
C2	0.5545 (4)	0.3442 (8)	0.99906 (18)	0.0582 (11)	
H2A	0.5431	0.2190	1.0174	0.070*	
C3	0.6420 (3)	0.3703 (7)	0.97159 (18)	0.0529 (10)	
C4	0.6630 (3)	0.5529 (7)	0.94406 (16)	0.0456 (8)	
C5	0.5892 (4)	0.7202 (7)	0.94479 (17)	0.0535 (10)	
H5A	0.6003	0.8463	0.9267	0.064*	
C6	0.5003 (3)	0.7029 (8)	0.9717 (2)	0.0582 (11)	
H6A	0.4525	0.8156	0.9721	0.070*	
C7	0.7577 (3)	0.5771 (6)	0.91542 (15)	0.0451 (8)	
C8	0.7929 (3)	0.4063 (7)	0.88590 (18)	0.0546 (10)	
H8A	0.7563	0.2761	0.8840	0.065*	
C9	0.8827 (3)	0.4311 (7)	0.85932 (18)	0.0545 (10)	
H9A	0.9059	0.3167	0.8400	0.065*	
C10	0.9376 (3)	0.6252 (7)	0.86148 (15)	0.0497 (9)	
C11	0.9034 (3)	0.7916 (7)	0.89094 (19)	0.0560 (10)	
H11A	0.9409	0.9208	0.8930	0.067*	
C12	0.8149 (3)	0.7706 (7)	0.91747 (17)	0.0526 (10)	
H12A	0.7931	0.8858	0.9369	0.063*	
C13	1.0342 (4)	0.6569 (9)	0.83342 (18)	0.0613 (12)	
C14	1.0706 (5)	0.4727 (13)	0.8035 (3)	0.089 (2)	
H14A	1.1369	0.5069	0.7908	0.133*	
H14B	1.0238	0.4486	0.7774	0.133*	
H14C	1.0749	0.3427	0.8229	0.133*	
F3	0.3553 (3)	1.0087 (6)	1.04498 (16)	0.0822 (11)	
F4	0.0411 (2)	1.2848 (4)	1.09719 (14)	0.0734 (9)	
O2	−0.3281 (3)	0.6449 (6)	1.2310 (2)	0.0821 (11)	
C15	0.2688 (3)	0.9868 (7)	1.0722 (2)	0.0526 (12)	
C16	0.1987 (4)	1.1521 (7)	1.07165 (18)	0.0580 (11)	
H16	0.2096	1.2788	1.0537	0.070*	
C17	0.1123 (3)	1.1255 (6)	1.09822 (17)	0.0500 (9)	
C18	0.0917 (3)	0.9397 (7)	1.12637 (16)	0.0450 (8)	
C19	0.1654 (3)	0.7758 (7)	1.12533 (19)	0.0538 (10)	
H19	0.1547	0.6481	1.1429	0.065*	
C20	0.2550 (4)	0.7983 (7)	1.0985 (2)	0.0578 (10)	
H20	0.3042	0.6883	1.0985	0.069*	
C21	−0.0043 (3)	0.9146 (6)	1.15438 (15)	0.0445 (8)	
C22	−0.0595 (3)	0.7189 (7)	1.15141 (18)	0.0540 (10)	
H22	−0.0362	0.6067	1.1315	0.065*	
C23	−0.1482 (3)	0.6900 (7)	1.17769 (17)	0.0537 (10)	
H23	−0.1837	0.5585	1.1752	0.064*	
C24	−0.1853 (3)	0.8548 (7)	1.20778 (15)	0.0465 (8)	
C25	−0.1292 (4)	1.0488 (8)	1.21097 (18)	0.0539 (10)	
H25	−0.1518	1.1606	1.2311	0.065*	
C26	−0.0409 (3)	1.0768 (7)	1.18471 (16)	0.0506 (9)	
H26	−0.0050	1.2078	1.1874	0.061*	
C27	−0.2822 (4)	0.8178 (8)	1.23485 (19)	0.0593 (11)	
C28	−0.3252 (4)	0.9976 (11)	1.2659 (2)	0.0748 (16)	
H28A	−0.3950	0.9646	1.2738	0.112*	
H28B	−0.3223	1.1340	1.2487	0.112*	
H28C	−0.2860	1.0089	1.2950	0.112*	

### Atomic displacement parameters (Å^2^)

**Table:**

	*U*^11^	*U*^22^	*U*^33^	*U*^12^	*U*^13^	*U*^23^
F1	0.0568 (18)	0.080 (2)	0.084 (2)	−0.0068 (13)	0.0293 (18)	−0.0080 (16)
F2	0.087 (2)	0.0644 (18)	0.094 (2)	0.0219 (15)	0.021 (2)	0.0117 (18)
O1	0.066 (2)	0.085 (3)	0.107 (3)	−0.003 (2)	0.025 (2)	0.009 (2)
C1	0.056 (3)	0.063 (3)	0.051 (3)	−0.0082 (19)	0.008 (2)	−0.011 (2)
C2	0.065 (3)	0.058 (2)	0.051 (2)	−0.005 (2)	0.009 (2)	−0.003 (2)
C3	0.051 (2)	0.049 (2)	0.059 (3)	0.0048 (16)	0.000 (2)	−0.008 (2)
C4	0.0427 (19)	0.0438 (18)	0.050 (2)	0.0015 (16)	−0.0015 (17)	−0.0036 (18)
C5	0.055 (2)	0.051 (2)	0.054 (2)	0.0113 (17)	0.0037 (19)	0.005 (2)
C6	0.043 (2)	0.066 (3)	0.066 (3)	0.0130 (18)	0.004 (2)	−0.004 (2)
C7	0.0453 (19)	0.0434 (18)	0.047 (2)	0.0043 (15)	−0.0014 (16)	−0.0040 (18)
C8	0.052 (2)	0.048 (2)	0.064 (3)	−0.0013 (17)	0.004 (2)	−0.013 (2)
C9	0.055 (2)	0.055 (2)	0.053 (2)	0.005 (2)	0.008 (2)	−0.009 (2)
C10	0.049 (2)	0.057 (2)	0.0430 (19)	0.0049 (17)	−0.0023 (17)	0.0005 (19)
C11	0.052 (2)	0.051 (2)	0.065 (3)	−0.0007 (18)	0.004 (2)	0.002 (2)
C12	0.055 (2)	0.0459 (19)	0.057 (2)	0.0044 (17)	0.008 (2)	−0.006 (2)
C13	0.055 (2)	0.071 (3)	0.059 (3)	0.009 (2)	0.004 (2)	0.016 (2)
C14	0.068 (4)	0.107 (4)	0.091 (5)	−0.005 (3)	0.036 (4)	−0.008 (4)
F3	0.070 (2)	0.090 (2)	0.086 (3)	−0.0063 (16)	0.028 (2)	−0.0042 (19)
F4	0.0778 (19)	0.0526 (13)	0.090 (2)	0.0200 (13)	0.0111 (16)	0.0100 (15)
O2	0.064 (2)	0.073 (2)	0.109 (3)	−0.0153 (18)	0.018 (2)	0.004 (2)
C15	0.039 (2)	0.063 (2)	0.056 (3)	−0.0044 (16)	0.009 (2)	−0.007 (2)
C16	0.066 (3)	0.050 (2)	0.058 (2)	−0.0070 (19)	0.004 (2)	−0.001 (2)
C17	0.052 (2)	0.0434 (19)	0.055 (2)	0.0069 (15)	0.0025 (19)	−0.0022 (18)
C18	0.0430 (19)	0.0475 (19)	0.044 (2)	0.0043 (16)	0.0007 (16)	−0.0034 (18)
C19	0.045 (2)	0.051 (2)	0.065 (3)	0.0059 (17)	0.0014 (19)	0.002 (2)
C20	0.048 (2)	0.061 (2)	0.065 (3)	0.0073 (19)	0.006 (2)	−0.005 (2)
C21	0.0419 (18)	0.0474 (19)	0.044 (2)	0.0023 (15)	−0.0027 (16)	−0.0017 (18)
C22	0.057 (2)	0.0421 (19)	0.063 (3)	0.0021 (16)	0.002 (2)	−0.0096 (19)
C23	0.051 (2)	0.048 (2)	0.062 (3)	−0.0059 (16)	−0.0004 (19)	−0.0085 (19)
C24	0.0408 (18)	0.0514 (19)	0.047 (2)	0.0007 (15)	−0.0013 (17)	0.0041 (17)
C25	0.052 (2)	0.057 (2)	0.053 (2)	0.001 (2)	0.0037 (19)	−0.013 (2)
C26	0.0461 (19)	0.050 (2)	0.055 (2)	−0.0055 (16)	0.0019 (18)	−0.0084 (19)
C27	0.050 (2)	0.069 (3)	0.058 (3)	−0.0011 (19)	0.000 (2)	0.002 (2)
C28	0.070 (3)	0.094 (4)	0.060 (3)	−0.005 (3)	0.025 (3)	−0.006 (3)

### Geometric parameters (Å, °)

**Table:**

F1—C1	1.345 (6)	F3—C15	1.366 (6)
F2—C3	1.335 (5)	F4—C17	1.347 (5)
O1—C13	1.221 (7)	O2—C27	1.219 (6)
C1—C2	1.382 (7)	C15—C20	1.375 (7)
C1—C6	1.372 (8)	C15—C16	1.364 (7)
C2—C3	1.385 (7)	C16—C17	1.359 (7)
C2—H2A	0.9300	C16—H16	0.9300
C3—C4	1.378 (6)	C17—C18	1.402 (6)
C4—C5	1.407 (6)	C18—C19	1.390 (5)
C4—C7	1.479 (6)	C18—C21	1.486 (5)
C5—C6	1.386 (6)	C19—C20	1.396 (6)
C5—H5A	0.9300	C19—H19	0.9300
C6—H6A	0.9300	C20—H20	0.9300
C7—C8	1.403 (5)	C21—C26	1.384 (5)
C7—C12	1.399 (6)	C21—C22	1.398 (5)
C8—C9	1.395 (6)	C22—C23	1.383 (6)
C8—H8A	0.9300	C22—H22	0.9300
C9—C10	1.387 (6)	C23—C24	1.394 (6)
C9—H9A	0.9300	C23—H23	0.9300
C10—C11	1.377 (6)	C24—C25	1.396 (6)
C10—C13	1.497 (6)	C24—C27	1.491 (6)
C11—C12	1.378 (6)	C25—C26	1.377 (6)
C11—H11A	0.9300	C25—H25	0.9300
C12—H12A	0.9300	C26—H26	0.9300
C13—C14	1.476 (9)	C27—C28	1.504 (8)
C14—H14A	0.9600	C28—H28A	0.9600
C14—H14B	0.9600	C28—H28B	0.9600
C14—H14C	0.9600	C28—H28C	0.9600
			
F1—C1—C2	118.8 (5)	C20—C15—F3	118.9 (4)
F1—C1—C6	118.0 (4)	C20—C15—C16	122.4 (4)
C2—C1—C6	123.2 (5)	F3—C15—C16	118.7 (5)
C1—C2—C3	116.4 (5)	C17—C16—C15	117.8 (4)
C1—C2—H2A	121.8	C17—C16—H16	121.1
C3—C2—H2A	121.8	C15—C16—H16	121.1
F2—C3—C4	118.9 (4)	F4—C17—C16	118.6 (4)
F2—C3—C2	116.9 (4)	F4—C17—C18	117.6 (4)
C4—C3—C2	124.2 (4)	C16—C17—C18	123.9 (4)
C3—C4—C5	116.2 (4)	C19—C18—C17	116.0 (4)
C3—C4—C7	123.1 (4)	C19—C18—C21	121.6 (4)
C5—C4—C7	120.7 (4)	C17—C18—C21	122.4 (3)
C6—C5—C4	122.0 (4)	C18—C19—C20	121.6 (4)
C6—C5—H5A	119.0	C18—C19—H19	119.2
C4—C5—H5A	119.0	C20—C19—H19	119.2
C1—C6—C5	118.0 (4)	C15—C20—C19	118.4 (4)
C1—C6—H6A	121.0	C15—C20—H20	120.8
C5—C6—H6A	121.0	C19—C20—H20	120.8
C8—C7—C12	118.3 (4)	C26—C21—C22	117.9 (4)
C8—C7—C4	120.9 (4)	C26—C21—C18	122.4 (3)
C12—C7—C4	120.7 (4)	C22—C21—C18	119.6 (4)
C9—C8—C7	120.3 (4)	C23—C22—C21	120.8 (4)
C9—C8—H8A	119.8	C23—C22—H22	119.6
C7—C8—H8A	119.8	C21—C22—H22	119.6
C10—C9—C8	120.4 (4)	C22—C23—C24	121.1 (4)
C10—C9—H9A	119.8	C22—C23—H23	119.5
C8—C9—H9A	119.8	C24—C23—H23	119.5
C11—C10—C9	119.2 (4)	C23—C24—C25	117.8 (4)
C11—C10—C13	119.2 (4)	C23—C24—C27	119.2 (4)
C9—C10—C13	121.7 (4)	C25—C24—C27	123.0 (4)
C12—C11—C10	121.4 (4)	C26—C25—C24	120.9 (4)
C12—C11—H11A	119.3	C26—C25—H25	119.5
C10—C11—H11A	119.3	C24—C25—H25	119.5
C11—C12—C7	120.5 (4)	C25—C26—C21	121.5 (4)
C11—C12—H12A	119.8	C25—C26—H26	119.2
C7—C12—H12A	119.8	C21—C26—H26	119.2
O1—C13—C14	122.0 (5)	O2—C27—C24	120.4 (5)
O1—C13—C10	120.1 (5)	O2—C27—C28	119.8 (5)
C14—C13—C10	117.8 (5)	C24—C27—C28	119.8 (5)
C13—C14—H14A	109.5	C27—C28—H28A	109.5
C13—C14—H14B	109.5	C27—C28—H28B	109.5
H14A—C14—H14B	109.5	H28A—C28—H28B	109.5
C13—C14—H14C	109.5	C27—C28—H28C	109.5
H14A—C14—H14C	109.5	H28A—C28—H28C	109.5
H14B—C14—H14C	109.5	H28B—C28—H28C	109.5

### Hydrogen-bond geometry (Å, °)

**Table:**

*D*—H···*A*	*D*—H	H···*A*	*D*···*A*	*D*—H···*A*
C12—H12A···F2^i^	0.93	2.46	3.369 (6)	167
C22—H22···F4^ii^	0.93	2.40	3.318 (6)	167

Symmetry codes: (i) *x*, *y*+1, *z*; (ii) *x*, *y*−1, *z*.

### Table 2 C—F···π interactions (Å, °)

**Table:**

C—F···Cg^a^	F···Cg	C···Cg	γ^b^	C—F···Cg
C1—F1···Cg(C7→C12)^iii^	3.8358	3.5919	27.36	69
C15—F3···Cg(C21→C26)^iv^	3.8814	3.5966	27.14	68

Notes: Cg^a^ = centre of gravity of the six-membered ring. γ^b^ = angle defined by a line connecting centre of gravity of the six-membered ring with H atom and the normal to the six-membered ring. Symmetry codes: (iii) x+1/2, 1-y, z ; (IV) x+1/2, -y, z.

### Table 3 π···π interactions (Å, °)

**Table:**

π···π contacts	Cg···Cg(Å)	α^a^(°)	β^b^(°)	Cg···Plane(Å)
Cg(C1→C6)···Cg(C7→C12)^iii^	3.8221	4.87	27.32	3.616
Cg(C15→C20)···Cg(C21→C26)^iv^	3.8284	5.68	23.57	3.642

Notes: α^a^ = angle between planes of two aromatic rings. β^b^ = angle between Cg···Cg line and normal to the plane of the first aromatic ring. Symmetry codes: (iii) x+1/2, 1-y, z ; (IV) x+1/2, -y, z.
